# Repair and regeneration: ferroptosis in the process of remodeling and fibrosis in impaired organs

**DOI:** 10.1038/s41420-024-02181-2

**Published:** 2024-10-02

**Authors:** Jiali Yin, Xinjun Xu, Ying Guo, Caiyu Sun, Yujuan Yang, Huifang Liu, Pengyi Yu, Tong Wu, Xicheng Song

**Affiliations:** 1 Department of Otolaryngology, Head and Neck Surgery, Yantai Yuhuangding Hospital, Qingdao University, Yantai, China; 2 Shandong Provincial Key Laboratory of Neuroimmune Interaction and Regulation, Yantai, Shandong China; 3 Shandong Provincial Clinical Research Center for Otorhinolaryngologic Diseases, Yantai, Shandong China; 4 Second Clinical Medicine College, Binzhou Medical University, Yantai, Shandong 264003 China; 5 Qingdao Medical College, Qingdao University, Qingdao, 266071 China; 6 Medical Research Center, The Affiliated Hospital of Qingdao University, Qingdao University, Qingdao, 266000 China

**Keywords:** Oncogenesis, Cell death, Skin diseases

## Abstract

As common clinical-pathological processes, wound healing and tissue remodelling following injury or stimulation are essential topics in medical research. Promoting the effective healing of prolonged wounds, improving tissue repair and regeneration, and preventing fibrosis are important and challenging issues in clinical practice. Ferroptosis, which is characterized by iron overload and lipid peroxidation, is a nontraditional form of regulated cell death. Emerging evidence indicates that dysregulated metabolic pathways and impaired iron homeostasis play important roles in various healing and regeneration processes via ferroptosis. Thus, we review the intrinsic mechanisms of tissue repair and remodeling via ferroptosis in different organs and systems under various conditions, including the inflammatory response in skin wounds, remodeling of joints and cartilage, and fibrosis in multiple organs. Additionally, we summarize the common underlying mechanisms, key molecules, and targeted drugs for ferroptosis in repair and regeneration. Finally, we discuss the potential of therapeutic agents, small molecules, and novel materials emerging for targeting ferroptosis to promote wound healing and tissue repair and attenuate fibrosis.

## Facts

- Post-injury regeneration and antifibrosis, which are accompanied by redox imbalance and lipid peroxidation, have become urgent challenges in clinical practice.
- The essence of ferroptosis is the occurrence of iron-dependent peroxidation of membrane phospholipids, followed by the formation of protein adducts and disruption of the plasma membrane.
- Ferroptosis is involved in various disorders, not limited to cutaneous wounds, cartilage and bone remodeling and organ fibrosis diseases.
- Endogenous molecules, compounds and novel materials that specifically target ferroptosis hold promise for improving disease outcomes.

## Open questions

- What are the potential mechanisms for regulating tissue remodeling by ferroptosis in different organs and systems?
- What are the respective effects of activating or inhibiting ferroptosis under different conditions on tissues after injury?
- What drugs or new materials are currently available to target ferroptosis to modulate the remodeling process?

## Introduction

The repair processes of regeneration and remodeling caused by injury and inflammatory stimulation remain the focus of research. Wound healing is the natural response of an organism to external or internal injury stimuli. Its mechanisms are dynamic and complex, involving numerous cells and tissues and four sequential, overlapping key phases: hemostasis, inflammation, proliferation, and remodeling [1]. Alterations in these phases can result in poor prognostic outcomes, typically manifesting as poor or excessive healing, prolonged chronic wounds, or hyperproliferative pathological fibrosis [2]. In injured organs, the repair process is often unsatisfactory because of tissue defects, chronic inflammation accumulation and other abnormal pathologies. Damaged tissues and organs are unable to regenerate completely, resulting in failure of the final “remodeling phase”, which is commonly characterized by chronic, prolonged wounds, pathological scarring and fibrosis in the organs.

Recently, post-injury regeneration and antifibrotic effects involving multiple tissues and organs have become challenges in clinical practice. According to statistics, 25% of the ~500 million diabetic patients worldwide have recurrent skin ulcers that break down and are difficult to heal [3]. Impaired wound repair due to other factors such as radiation, UV light, chronic inflammation, and reactive oxygen species (ROS), is a growing public health problem [4]. Moreover, fibrosis that develops in the parenchymal organs (heart, liver, kidney, and lungs) stimulated by injury results in gradual deterioration caused by several chronic diseases [5]. The current lack of very effective mechanisms and tools is intimately related to the complex pathogenesis of impaired organs. Therefore, exploring the core factors and key mechanisms of the post-injury process and developing new drugs and methods for targeted therapy are important.

**Fig. 1:**
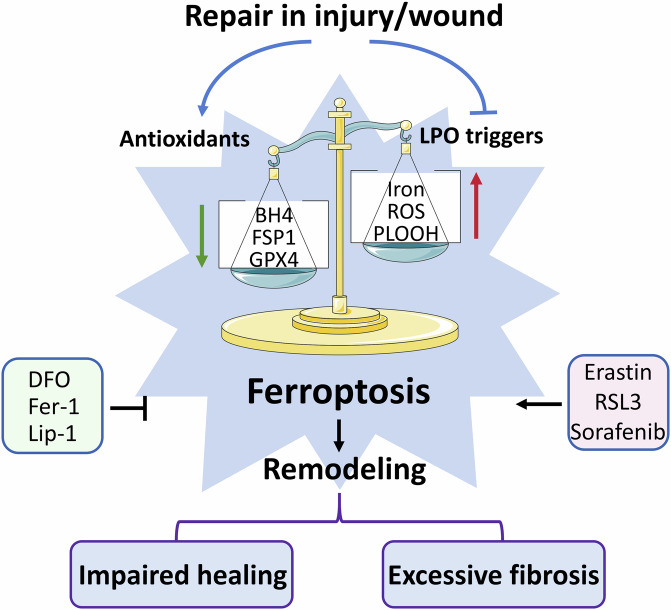
Schematic of ferroptosis caused by injury or wound. Injury triggers the process of lipid peroxidation (LPO) and attenuates inhibitory mechanisms preventing LPO. The disruption of redox homeostasis induces ferroptosis, which, with other factors, may result in impaired healing or excessive fibrosis.

Initiated by gene regulation following stimulation by internal and external environments, programmed cell death (PCD), including apoptosis, autophagy, pyroptosis, necroptosis, ferroptosis, and cuproptosis, contributes to maintaining physiological homeostasis and repairing damage in organisms [6]. Due to the fact that apoptosis and autophagy were identified earlier, current studies have reported more on the role of apoptosis and autophagy in wound healing. Ferroptosis, a novel mode of PCD defined in 2012 [7], is characterized by intracellular iron overload and the accumulation of iron-dependent lipid peroxides. Additionally, iron metamorphosis results in oxidoreductase inhibition, particularly by the lipid peroxide scavenger glutathione peroxidase 4 (GPX4) [8]. Ferroptosis differs morphologically from apoptosis or necrosis and does not involve the cellular machinery that mediates apoptosis or necroptosis [9]. However, the specialized iron homeostasis dysregulation, redox imbalance, and lipid metabolism abnormalities in ferroptosis are critical for cell and animal survival, especially in pathological processes such as various acute injuries, chronic inflammatory trauma, and degenerative changes due to ageing. Extensive research has been conducted on the pathological role of ferroptosis in regulating iron disorders, ROS, and lipid peroxidation products in skin wound disorders, bone and joint remodeling, and multiorgan fibrosis. The relationship between ferroptosis and injury repair is shown in Fig. 1. In this review, we focus on the relevance of post-injury repair and regeneration-related diseases to ferroptosis on a pathological basis, providing potential therapeutic targets and ideas. We also highlight various novel drugs, small molecules, and materials that have been applied in preclinical studies to target ferroptosis in various diseases and used in relevant disease models.

## Overview of ferroptosis

**Fig. 2:**
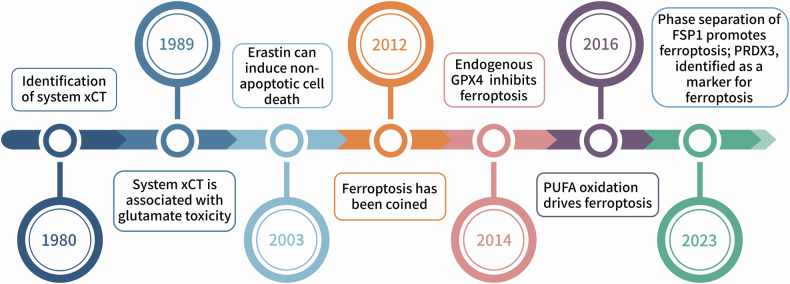
Development of ferroptosis. Overview of ferroptosis development timeline.

From the discovery of nonapoptotic cell death induced by erastin and RSL3 [10, 11], which was formally coined “ferroptosis” by Dixon et al. [7], to the breakthrough identification of the specific marker of ferroptosis, peroxiredoxin PRDX [12], the development of ferroptosis (Fig. 2) has led to various debates and explorations by researchers. The distinctive morphological changes associated with ferroptosis include mitochondrial shrinkage, a reduction in or disappearance of mitochondrial cristae, and increased mitochondrial membrane density [13]. With respect to metabolic and regulatory mechanisms, cells undergoing ferroptosis sustain changes in iron metabolism, lipid peroxidation formation, ROS production, and the glutathione/FSP1 antioxidant system. Ferroptosis is the iron-dependent peroxidation of membrane phospholipids, followed by protein adduct formation and plasma membrane disruption. When cellular iron metabolism is disturbed, free iron over accumulates in cells, which generates ROS via the Fenton reaction [14]. ROS further catalyze the lipid peroxidation of unsaturated fatty acids that are highly expressed in the cellular membranes, which leads to the rupture of cellular membranes and the accumulation of lipid peroxides, resulting in cell death [15].

**Fig. 3:**
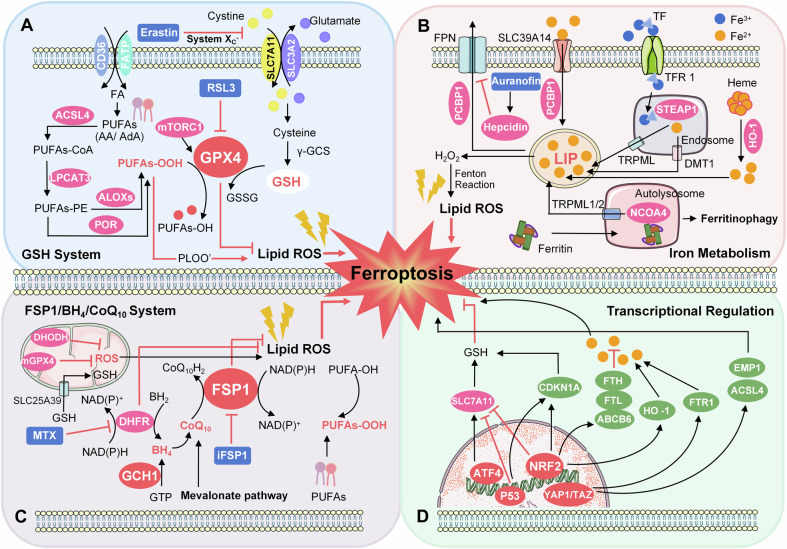
The main regulatory mechanisms of ferroptosis. **A** Cellular antioxidant systems eliminating lipid peroxidation of membrane phospholipids mainly function through the GSH system. ACSL4 and LPCAT3 are required for the production of PUFAs-PE. Activation of ALOXs, PORs, or NOXs promotes lipid peroxidation. **B** Iron metabolism has been associated with ferroptosis. Generally, excess iron is stored in ferritin, while free iron, present as part of the LIP, accumulates due to metabolic imbalance. It has been shown that NOCA4 is a cargo receptor that binds to ferritin and delivers it to autolysosomes, thereby releasing free iron. Free iron is involved in the generation of ROS through the Fenton reaction. **C** The CoQ10/FSP1 and GCH1/BH4/DHFR metabolic pathways can also eliminate lipid peroxidation of membrane phospholipids. In mitochondria, DHODH and mGPX4 are involved in scavenging ROS. **D** Several key transcriptional regulators such as Nrf2, p53, ATF4, SP1, HIF-1A, and YAP/TAZ have been evidenced to modulate ferroptosis.

We summarize the relevant pathways and regulatory mechanisms of ferroptosis in Fig. 3, respectively. The representative inducers and inhibitors that target ferroptosis are summarized in Table 1. The inflammatory response and ROS accumulation following injury and irritation naturally associate tissue regeneration and remodeling processes with ferroptosis, which we comprehensively address below.

**Table 1:** Representative ferroptosis inducers and inhibitors.

Reagent	Effect	Target	Main mechanism	Reference
BSO	Induction	GSH	GSH depletion, lipid peroxidation	[235]
Cisplatin	Induction	GSH	GSH depletion, ROS accumulation	[202]
Erastin	Induction	System Xc^-^	GSH depletion, GPX4 inactivation, lipid peroxidation	[185]
IFN-γ	Induction	System Xc^-^	lipid peroxidation, GSH depletion	[236]
Sorafenib	Induction	System Xc^-^	GSH depletion, ROS accumulation, lipid peroxidation	[195]
Sulfasalazine	Induction	System Xc^-^	GSH depletion, SLC7A11 downregulation	[12]
RS3	Induction	GPX4	GPX4 downregulation, ROS accumulation, lipid peroxidation	[210]
Statins (simvastatin, lovastatin, and pravastatin)	Induction	GPX4	GPX4 downregulation, lipid peroxidation	[237]
Withaferin A	Induction	GPX4	GPX4 downregulation, lipid peroxidation	[238]
Neratiniba	Induction	Fe	Iron activation, ROS accumulation	[239]
Lapatinib	Induction	Fe	Iron activation, ROS accumulation	[240]
Deferoxamine	Inhibition	Fe^2+^	Chelates intracellular iron	[39]
Ferrostatin-1	Inhibition	ROS	Prevents ROS generation	[85]
Liproxstatin-1	Inhibition	ROS	Prevents lipid peroxidation	[221]
XJB-5-131	Inhibition	ROS	Eliminates poisonous ROS	[163]
Vitamin E	Inhibition	5-Lipoxygenase	Inhibits PUFAs catalyzing into hyperoxide	[241]
Zileuton	Inhibition	5-Lipoxygenase	Inhibits lipid peroxidation	[163]

## Role of ferroptosis in cutaneous wound healing and regeneration

A sustained inflammatory response during cutaneous wound repair is closely related to postoperative outcomes. Delayed wound healing can result in chronic wounds, often leading to an economic burden and social disorders in patients [16]. Ferroptosis is involved in the occurrence, development, treatment, and prognosis of several types of wounds [4]. Extensive research on ferroptosis may provide new insights into improving and promoting wound healing.

### Iron overload and ferroptosis in diabetic wounds

Diabetes is a metabolic disease that affects human health, and its annual prevalence is expected to surpass 700 million by 2045 [17]. In a persistent hyperglycemic (HG) environment, the skin of patients with hyperglycemia is highly susceptible to damage and recurrent infections. The wounds exist in a prolonged phase of the hyperinflammatory response, which manifests as ulcers, deep injuries, insufficient local blood supply, and delayed healing. Severe cases of poorly treated diabetic wounds are often at risk of osteomyelitis, amputation, and death, and exploration of the underlying mechanisms and development of targeted treatment programs are urgently needed.

Persistent oxidative stress is closely related to the delayed healing of diabetic wounds, and lipid peroxide accumulation via the circulation further contributes to iron metabolic pathway disruption. The serum ferritin concentration is positively correlated with type 2 diabetes mellitus [18], and patients with diabetes have high ferritin and various free iron serum levels [19]. These factors can impair wound healing and inversely interfere with diabetes development by reducing insulin secretion and causing mitochondrial dysfunction [20]. The increase in oxidative stress caused by the combined stimulation with HG and iron overload exacerbates endothelial dysfunction [21]. GPX4 and SLC7A11 expression in diabetic mouse renal tissues was lower than that in control mouse renal tissues, while the TFR1 expression was elevated. Following Fer-1 treatment in the high glucose group, the concentrations of lipid peroxides and iron, which were originally highly expressed, were significantly decreased [22]. Ferroptosis activation in fibroblasts and vascular endothelial cells stimulated by oxidative stress under HG conditions results in reduced cell activity and impaired migration. However, iron deficiency in diabetic wounds delays extracellular matrix (ECM) deposition, whereas exogenous iron supplementation promotes collagen type I and III deposition [23], which may be related to the polarization of M2 macrophages [24]. Ferroportin (FPN) deficiency inhibits angiogenesis and macrophage proliferation during wound healing [25]. The precise control of iron homeostasis is essential for maintaining metabolic balance in the body [26]. Whether the relationship between ferroptosis and diabetes-related damage is causal, concomitant, or consequential remains unclear.

Macrophages, vascular endothelial cells, fibroblasts, and keratinocytes are modulated by ferroptosis during the inflammatory, proliferative, and remodeling phases to perform reparative functions [27]. Zhou et al. [28] reported that iron overload could activate p53 acetylation via the excessive release of ROS and M1 macrophage polarization. Macrophage polarization can also be regulated by HMGB1, which is induced by ferroptosis to promote wound closure [29]. During the inflammatory phase, iron overload in the vascular endothelial cells enhances ROS accumulation, which recruits monocytes to the periwound area to promote their differentiation into macrophages [30]. Following treatment with the ferroptosis inhibitor Fer-1, nitric oxide synthase expression in mouse endothelial cells increases, reducing immune cell adhesion and alleviating inflammation [31]. In vitro experiments have confirmed that HG exacerbates lipid peroxidation in endothelial cells and downregulates GPX4 expression to promote cell death, which is reversed by the inhibitors DFO and Fer-1 [32]. Low levels of vascular endothelial growth factor (VEGF) in endothelial cells under HG conditions inhibit fibroblast migration and increase ROS and mitochondrial ROS production in both cell types, resulting in lipid peroxide accumulation. However, in vivo and in vitro experiments confirmed that Fer-1 reversed these effects and promoted wound healing in diabetic rats [22].

Ferroptosis in keratinocytes has not been clearly investigated in diabetes, but reduced levels of TNF-α and IL-6 have been observed in psoriasis keratinocytes [33]. The ferroptosis gene TP53 may play a critical role in diabetic foot ulcers [34], and MAPK3 may serve as a biomarker of ferroptosis, providing a novel direction for targeted therapy. Heme oxygenase-1 (HO-1), a pivotal mediator of ferroptosis, exerts anti-inflammatory and antioxidant effects and reduces the expression of inflammatory factors [35]. Its time-dependent downregulation under HG conditions inhibits collagen synthesis and fibroblast migration [36].

In combination with novel materials, ferroptosis inhibitors have promising applications in diabetic wound treatment. The novel DFO drug delivery system can effectively overcome the challenges of drug toxicity and short half-life, promote angiogenesis, and increase skin healing rates in diabetic mice [37]. Combining DFO with hydrogel nanoscaffolds, bioglass containing silica ions, and adipose-derived stem cells can effectively increase the upregulation of hypoxia-inducible factor (HIF) and VEGF and improve therapeutic effects [38, 39]. Additionally, noncoding RNA-circ-ITCH in the exosomes of bone marrow-derived stromal stem cells enhanced the angiogenic capacity of human umbilical vein endothelial cells and accelerated wound healing in mice with diabetic foot ulcers by alleviating ferroptosis under HG conditions [40]. These findings have resulted in novel therapeutic strategies for treating refractory diabetic ulcers.

### Irradiated and UV-driven wounds

Tumor radiotherapy and accidental irradiation are common triggers of clinical radiation-induced skin injuries (RISIs), and approximately 85–95% of patients experience varying degrees of skin injury following radiotherapy [41]. Excessive ROS production in localized skin induced by radiation results in cellular ferroptosis. Focusing on multiple ROS regulatory pathways (e.g., Keap1/Nrf2 and GCH1/BH4) can effectively improve the prevention and treatment of RISIs [42–44]. Local injection of rat plasma-derived exosomes significantly affected cutaneous wounds on irradiated rat backs. In vitro experiments revealed that this treatment inhibited ferroptosis in irradiated fibroblasts and promoted collagen production and ECM remodeling [45]. Other studies have confirmed that radiation damage induces ferroptosis, with lung adenocarcinoma and cervical cancer cells displaying increased susceptibility to ferroptosis due to irradiation [46] and the accumulation of ROS in breast cancer and melanoma cells [47]. Additionally, skin damage results from overexposure to UV light (UVA and UVB). Excessive UV irradiation induces lipid peroxide accumulation and ferroptosis in human skin keratinocytes [48], the accumulation of the oxidized PUFA phosphatidylethanolamine (PUFA-PE) is a critical factor. Feng et al. [48] reported that impaired GPX4 expression in human keratinocytes under UV irradiation caused intracellular iron overload, whereas the nicotinamide mononucleotide-dependent GPX4 pathway reversed this ferroptosis-related phenomenon.

Skin with delayed healing develops an imbalance in redox capacity owing to radiation or excessive UV irradiation, triggering ferroptosis. Antioxidant drugs may be effective in treating these conditions.

### Wound-related fibrotic skin disorders

#### Pathological scars

Abnormal wound healing processes following skin trauma can result in pathological scarring, such as hyperplastic scarring and keloids. Compared with hyperplastic scars, keloid scars are characterized by a stronger inflammatory response, mechanical tension, and excess collagen deposition than hyperplastic scars, posing a challenge for clinical treatment and requiring radical therapeutic approaches [49, 50]. Pathological scar formation is closely associated with ROS and lipid peroxidation [51]. Photodynamic therapy based on 5-aminolevulinic acid (5-ALA-PDT) is an effective treatment that generates ROS to exert cytotoxic effects [52, 53].

Following 5-ALA-PDT treatment, mRNA sequencing revealed an increase in AXSL4 expression, a key molecule involved in lipid accumulation during ferroptosis in keloid fibroblasts. Conversely, the expression of GPX4, GSS, and FTH1, which are negatively correlated with ferroptosis, decreased. Increased ROS and malondialdehyde (MDA) levels are inhibited by Fer-1; thus, the therapeutic effect of keloid treatment utilizing 5-ALA-PDT is achieved by increasing ferroptosis in fibroblasts [54]. Moreover, 11 ferroptosis-associated hub genes were identified and confirmed in keloid fibroblasts as exploitable markers for keloid diagnosis and treatment [55].

#### Systemic sclerosis (SSc)

Systemic sclerosis (SSc) is a chronic rheumatic disease characterized by immune dysfunction, inflammation, fibrosis, and skin and visceral vascular lesions [56, 57]. Despite significant breakthroughs in treating organ complications, therapeutic approaches are needed to prevent the progression of skin and visceral organ fibrosis progression [58]. Recent bioinformatic studies at the single-cell transcriptome level have revealed aberrant ferroptosis in multiple sclerosis, SSc-associated interstitial lung disease, and proinflammatory factors that can drive changes in ferritin deposition [59]. However, the molecular mechanisms underlying these responses remain unclear.

In SSc mice with increased ACSL4 expression, ferroptosis is present and occurs in both skin and lung tissues, and ACSL4 inhibition effectively prevents fibrosis progression and provides protection from the inflammatory milieu. ACSL4 induces ferroptosis in inflammatory macrophages and exacerbates fibrotic progression; therefore, ACSL4 is a potential therapeutic target [60]. More research is needed to connect inflammation and ferroptosis to elucidate the mechanisms involved in the ferroptosis response.

### Inflammatory skin diseases

Various inflammatory skin diseases are attributed to the disruption of the skin barrier and aberrant structural components [61], and ferroptosis actively affects the epidermal barrier and skin homeostasis. Selenium deficiency affects GPX4 biosynthesis, and selenium levels are decreased in patients with psoriasis with longer disease durations and are correlated with psoriasis severity, which may explain the reduced antioxidant activity and susceptibility to ferroptosis in patients with psoriasis [62]. Keratinocyte-specific GPX4 knockout mice exhibit inflammatory cell infiltration and altered appendage morphology in the dermis, and the keratinocytes exhibit ferroptosis characterized by peroxidation [63]. ALOX15 is an enzyme involved in ferroptosis that maintains skin homeostasis and plays dual roles. The ALOX15 metabolic pathway regulates macrophages to promote anti-inflammatory mediator synthesis [64], and ALOX15 inhibition contributes to alleviating inflammatory cell infiltration during contact and atopic dermatitis [65]. The intrinsic mechanism underlying ALOX15 involvement in ferroptosis to maintain skin homeostasis requires further investigation.

Recently, the medical community has interpreted psoriasis as a systemic inflammatory disease with multiple skin manifestations, including immunity-related and multisystem involvement [66]. Oxidative stress and various T cell subsets are essential for psoriasis pathogenesis [67]. Arbiser et al. [68] reported that psoriatic lesions expressed high Nrf2 levels and low GPX4 levels. Single-cell RNA sequencing revealed that lipid peroxidation in keratinocytes results in abnormal activity of the Th22/Th17 pathway [33]. The levels of ROS and AA metabolites, key factors in ferroptosis, are also elevated in patients with psoriasis [69]. Particularly, increased MDA, which is strongly associated with ferroptosis, was assessed as an oxidative stress marker highly correlated with psoriasis severity [70]. Mitochondrial alterations during ferroptosis were observed in the keratinocytes of mice with imiquimod-induced psoriasis. Topically applying Fer-1 could decrease lesion severity; however, ferroptosis agonists did not exacerbate the lesions. Thus, ferritin deposition may not be an initiating factor in psoriasis induction but rather an important link in the pathogenesis that exacerbates the inflammatory response [33]. Additionally, alterations in Treg function, phenotype, and differentiation are important pathogenic mechanisms in psoriasis [71]. Treg cells rely on the GPX4 pathway to resist cellular ferroptosis to maintain normal mitochondrial homeostasis and the expression of inflammatory factors such as IL-1β [72].

## Ferroptosis with cartilage and bone remodeling

### Osteoarthritis

Osteoarthritis (OA) is a multifactorial degeneration of the entire joint, that affects more than 303 million people worldwide, particularly in the elderly population [73, 74]. Joint replacement surgery is the conventional treatment for end-stage OA; however, its results are limited by the artificial joint lifespan [75]. Oxidative stress plays a crucial role in the pathological processes of OA (cartilage degeneration, bone loss, sclerosis, synovial inflammation, and hyperplasia) [76]. Exploring the therapeutic potential of lipid peroxidation and chondrocyte ferroptosis in OA is promising.

#### Chondrocyte ferroptosis in cartilage degeneration in OA

Ferroptosis in chondrocytes plays an important role in OA progression and provides potential insights into its treatment. Chondrocytes are stimulated by IL-1β and ferric ammonium citrate (FAC) to undergo induced ferroptosis, accompanied by ROS accumulation and changes in related molecules, such as GPX4, ACSL4, and P53 [77]. The GSH/GPX4 pathway has been extensively investigated in OA pathogenesis. GPX4-dependent ferroptosis in chondrocytes regulates ECM degradation via the MAPK/NF-κB signaling pathway [78]. Moreover, as identified by single-cell RNA-seq analysis, revealed that TRPV1 activation attenuates chondrocyte ferroptosis by restoring GPX4 expression [79]. Mechanical stress exacerbates OA [80], and recent studies have shown that calcium ion influx in Piezo1 channels caused by mechanical stress overload accelerates chondrocyte ferroptosis. Conversely, FSP1 and coenzyme Q10 alleviate OA progression in GPX4-deficient mice [81].

The relationship between oxidative stress injury and ferroptosis in OA has been reported; in particular, oxidative stress and mitochondrial dysfunction in chondrogenic degeneration can be caused by the Fenton reaction induced by iron overload [77]. ROS and lipid peroxide accumulation in chondrocytes increases under high-dose FAC stimulation, promotes chondrocyte apoptosis, and inhibits type II collagen synthesis [82]. IL-1β, a cytokine for establishing an in vitro model of OA [83], can induce osteoclast (OC) catabolism by disrupting iron homeostasis by promoting iron influx and increasing matrix metalloproteinase (MMP) expression [84]. Wang et al. [85] found that IL-1β stimulation increased the iron content within both chondrocytes and mitochondria. OA exacerbation is due to the promotion of ferroptosis and ferritin autophagy mediated by NCOA4 [86], and IL-1β can further influence iron homeostasis via NCOA4. DFO, an iron chelator, effectively suppresses type II collagen cleavage in osteoarthritic cartilage and reduces disc degeneration by inhibiting ferroptosis [87]. GPX4, oxidative stress, lipid metabolism, iron content, and other factors can be targeted to control ferroptosis in chondrocytes, thereby inhibiting the inflammatory response and cartilage degeneration in OA.

#### Ferroptosis promotes synovial inflammation and bone loss

Repeated synovium involvement in OA development results in synovial inflammation, and synovial cell ferroptosis in rheumatoid arthritis has been reported [88]. Synovial hyperplasia in other joint diseases can be caused by iron overload [89]. Ferroptosis-related genes (IL-6, IL-1B, EGR1, and ATF3) can be used as biomarkers of synovial tissue hyperplasia in OA [90], and macrophages are active in synovitis inflammation in OA [91]. Therefore, future studies on OA may involve the selection of macrophages and synoviocytes to explore ferroptosis. Subchondral bone loss and advanced osteosclerosis are important pathological alterations in OA [92]. He et al. [93] reported that subchondral bone loss occurred in an OA model with iron overload. Ferroptosis occurs in osteoblasts (OBs) in an iron-overloaded environment, while bone resorption by OCs is increased, accelerating bone loss, which can be rescued by the antioxidant NAC [93] and the iron chelator DFO [94]. Ferroptosis is essential for homeostasis and subchondral bone and synovium structural integrity and could provide an alternative strategy for managing OA in clinical settings.

### Osteoporosis

The incidence of osteoporosis (OP) is increasing with the aging population. Decreased bone mass and microarchitecture destruction result in increased bone fragility in patients with OP [95], and bone remodeling is caused by an imbalance between bone formation and resorption [96]. The primary functional cells involved in neobone formation are OBs, which synthesize and release a type I collagen-rich ECM and participate in collagen fiber deposition. Conversely, OC-mediated bone resorption is an important bone metabolism process. Among the MMPs, cathepsin K consumes minerals and collagen from bone cavities. Thus, at the cellular level, bone remodeling is regulated by the mutual constraints of OBs and OCs [97].

OBs and OCs contain key molecules that regulate their functions. Runx2, a transcription factor in OBs, regulates the osteoblast protein and osteocalcin (OCN) in the bone matrix and influences the interactions of signaling pathways such as the Wnt, MAPK, BMP, and Notch pathways to regulate bone formation [98, 99]. The regulation of OCs by the RANK/RANKL or JAK2/STAT3/RANKL axis stimulates their proliferation and differentiation, which affects their bone phagocytic function and causes bone loss [100–102]. Recently, researchers have committed to precisely controlling bone remodeling by regulating PCD to prevent and treat OP.

#### Ferroptosis in the bone remodeling in OP

Disruption of calcium homeostasis in OP involves fluctuations in iron levels. Zhang et al. [103] found that lower serum calcitonin levels were accompanied by significantly elevated serum iron levels in patients with OP, suggesting that iron overload is a risk factor for OP. Additionally, iron overloads the resulting Fenton reaction, which catalyzes the excess formation of ROS and the accumulation of lipid peroxides. Excessive oxidative stress and iron dependence are hallmark features of ferroptosis, in which high levels of ROS generation impair important components of the organism.

The accumulation of ROS is also an essential factor in the pathogenesis of OP [104], and excess ROS reverse the direction of the normal process of bone remodeling, resulting in bone loss and reduced bone synthesis. The role of ferroptosis in the regulation of OBs and OCs, which are critical for the balance between bone formation and resorption in OP, is becoming a point of focus. NRF2/HO-1 signaling pathway activation effectively mitigates oxidative stress and scavenges ROS by decreasing the ferroptosis of MC3T3-E1 cells, and in increasing bone formation [105]. Ferroptosis in OBs is also induced by the activation of the METTL3/ASK 1-p38 signaling pathway [106] as well as by Nrf2-ARE, which is downregulated by the effects of CoCrMo nanoparticles [107]. Mitochondrial pathways are closely associated with redox reactions. Mitochondrial ferritin (FtMt), which is involved in OB ferroptosis, exerts protective effects by storing iron and ferrous ions [108]. Silencing FtMt triggers mitochondrial autophagy-dependent ferroptosis in OBs via the ROS/PINK1/Parkin pathway [109]. Elevated AGE levels in patients with OP inhibit OB proliferation and differentiation by inducing ferroptosis [110]. Moreover, iron overload suppresses the cellular activity of MC3T3-E1 and OB cells with increased apoptosis, and the PI3K/AKT and FOXO3a/DUSP14 pathways are protective mechanisms in response to oxidative stress in OP [111].

Conversely, iron overload and ferroptosis are positively correlated with OC differentiation and bone loss. Iron overload results in reduced OCN content and bone density and increases the levels of the inflammatory factors IL-6 and ROS in OBs [112]. Bone resorption is promoted by ferritin autophagy and RANKL-induced ferroptosis in OCs. Flavonoid glycosides that target RANKL can prevent OP by inhibiting ROS synthesis [100], making RANKL a candidate for future therapeutic targeting.

Additionally, OC differentiation and activity can be inhibited by heparin and bisphosphonates [103, 113], both of which have potential therapeutic mechanisms related to ferroptosis limitations. OP treatment and prevention could be alternatively regulated using noncoding RNAs, and miR-3074-5p inhibition in MC3T3-E1 cells attenuates cell death during iron overload by acting on its target gene, Smad4 [111]. Additionally, the lncRNA XIST is upregulated in a high-iron-ion environment to regulate OB apoptosis via the XIST/miR-758-3p/caspase3 axis [114]. Glucocorticoid-induced inhibition of osteogenesis in OP can be reversed by endothelial cell exosomes via ferritin autophagy-dependent ferroptosis [115, 116]. With the refinement of the regulatory network mechanism between ferroptosis and bone remodeling, more theoretical rationales for the prevention and treatment of OP will be forthcoming.

## Ferroptosis in other tissues and organ remodeling

### Regulatory mechanism of ferroptosis in vascular remodeling diseases

#### Suboptimal spiral artery remodeling in preeclampsia (PE)

PE is a placenta-associated disease characterized by hypertension with the damage to maternal end organs, and is one of the major contributing factors to both maternal and fetal mortality and morbidity [117]. Recently, increasing evidence ha suggested that the pathogenesis of PE is related to ferroptosis of trophoblasts, which might cause aberrant pathologies such as inflammation accumulation, hemodynamic disorders and suboptimal spiral artery remodeling [118, 119].

An increasing number of studies have suggested that ferroptosis might cause defective remodeling of spiral arteries by inhibiting the cellular differentiation of trophoblast cells, activating excessive inflammatory responses, and affecting hemodynamics. Differentiation of cytotrophoblast cells into either syncytiotrophoblast cells or extravillous trophoblast cells is crucial for proper spiral artery remodeling [120]. The exposure of RSL3, a ferroptosis inducer, is associated with the diminished production of hormones required for the differentiation of the cytotrophoblasts [121]. RSL3, which are two crucial molecules involved in trophoblast differentiation, has been reported to cause the accumulation of PUFA-PLs and insufficient cAMP and PKA in trophoblasts [122]. Moreover, a diminished ferroptosis activation threshold might be present in trophoblast cells with increased vulnerability in the presence of low-dose exposure to RSL3 [123]. In PE patients, diminished GPX4 levels in the placenta promote the release of proinflammatory DAMPs [124]. Therefore, a vicious cycle involving ferroptosis, inflammation and aberrant hemodynamics can be proposed in the pathology of PE [125]. Ferroptosis is involved in the pathogenesis of suboptimal spiral artery remodeling, leading to aberrant hemodynamics in the maternal fetal circulation. This hemodynamics subsequently contributes to the risk of ischemia reperfusion injury and redox imbalance, which leads to the susceptibility of trophoblasts to ferroptosis and triggers inflammation. The treatment and development of targeted medications for these synergistic factors need to be further explored.

#### Ferroptosis in vascular remodeling associated with atherosclerosis

Atherosclerosis refers to the build-up of plaque in arteries, which is composed of a distinct core and outer layer. The adaptive vascular remodeling within the atherosclerotic wall can be either constrictive or expansive [126]. Constrictive remodeling is characterized by inflammation and matrix breakdown, leading to fibroplasia and further narrowing of the lumen. In contrast, expansive remodeling involves vasodilation to compensate for the narrowing of the lumen caused by plaque. Patients with expansive remodeling have been reported to have plaques that are more prone to rupture, leading to acute coronary syndrom [127]. Iron metabolism plays play a critical role in the development and progression of atherosclerotic plaques. Aberrant iron metabolism contributes to ROS overproduction, macrophage polarization, and influences the production of IL-1β, a key regulator of thrombosis, all of which are important in regulating plaque size and stability [128–130].

Growing evidence confirms that ferroptosis is associated with the development of atherosclerosis and can be an effective therapeutic target. At the cellular level, prenyldiphosphate synthase subunit 2 (PDSS2) blunted ferroptosis by activating Nrf2 to inhibit iron accumulation and reduce ROS production [131]. In ApoE^−^/^−^ mice models, Inhibition of ferroptosis and lipid peroxidation by GPX4 and Fer-1 use further attenuated atherosclerosis [31, 132]. Collectively, iron metabolism and ferroptosis contribute to the pathogenesis of atherosclerosis, which are promising therapeutic targets that need further investigation. Effective targeting of ferroptosis for intervention against cells critical for constrictive and expansive of the remodeling process is also a further research claim.

### Ferroptosis in the remodeling of central nervous injuries and neurodegenerative disease

#### Traumatic brain injury (TBI) and spinal cord injury (SCI)

TBI and SCI are the primary causes of traumatic injuries to the central nervous system (CNS), with the increased incidence given the growing and aging population. Patients with TBI and SCI require a prolonged and high level of specialized nursing, causing long-term burdens [133]. Inflammatory edema formed after primary SCI causing acute injury to neurons secondary to a cascade effect, forming a remodeled fibrotic scar inside the cystic cavity leading to progressive neuronal and glial cell death and inhibition of axonal regeneration [134]. Secondary injury in TBI causes increased intracranial pressure which blocks blood flow and further leads to oxidative damage to brain tissue [135]. Recent evidence suggests that ferroptosis is critical for secondary injury after TBI and SCI. Investigating the mechanisms of ferroptosis may deepen the understanding of the pathophysiological mechanisms of TBI and SCI and help guide strategies to improve neuronal regeneration and acute and chronic functional recovery.

Ferroptosis is universal in the nervous system, from neurogenesis to degeneration and from acute injury to chronic prognosis. A significant increase in Fe^3+^ and total iron levels can be detected in serum on day 1 after TBI. Fe^2+^, Fe^3+^ and total iron increase in the cortex up to 3 days post-trauma and continue to increase over time [136]. Atrophied mitochondria can be observed at the edges of the injured cortex by transmission electron microscopy [137]. TBI rapidly leads to ferroptosis, with increased ALOX15 and ACSL4 expression and decreased GSH in the ipsilateral cerebral cortex [138]. Similar to TBI, mitochondrial atrophy can be observed 15 min after SCI, with a significant enhancement at 24 h. Total iron levels and lipid peroxidation are increased, and GPX4, SLC7A11 and GSH are progressively depleted within 2 weeks after injury [139, 140].

Accumulation of iron, lipid, ROS and associated microglia/macrophage transformation form a link between iron metaplasia and neuroinflammation [141]. Microglia and macrophages may polarize to a deleterious (M1) or beneficial (M2) state, with a predominant shift to the M1 phenotype after CNS trauma. NADPH oxidase 2 (NOX2), a biomarker of ferroptosis, markedly increased post-TBI in the ipsilateral cortex, especially in the microglia and macrophages at 4 and 7 days [137, 142]. As for other core genes in ferroptosis, knockdown of ACSL4 inhibits microglia activation and subsequent production of proinflammatory cytokines, whereas inhibition of sirtuin 2 increases p53 expression and acetylation, exacerbating ferroptosis after TBI [143, 144].

Recent studies have listed some medicines for inhibiting ferroptosis after CNS traumatic injuries to help repairment. Iron chelator deferoxamine can promote nerve repair and restore long-term motor function by inhibiting gliosis through increased GPX4 expression and attenuated iron overload [139, 145]. Fer-1, an inhibitor for ferroptosis, suppressed ferroptosis in oligodendrocytes and activation of reactive astrocytes and microglia, ultimately attenuating white matter damage and improving functional recovery in a rat model of SCI [146]. As in TBI, treatment with Fer-1 reduced intracellular iron accumulation and the number of degenerating neurons, reduced the size of damaged lesions, and improved prolonged motor and cognitive prognosis in mice [136]. Some antioxidant activators may also be effective in targeting ferroptosis after TBI and SCI. SRS 16-86, proanthocyanidin and zinc gluconate have all been reported to increase GPX4 and system Xc- and effectively reduce lipid oxidation, gliosis and neuronal damage in SCI models [146–148]. Lip-1 and Melatonin both prevented TBI-induced decreased cortical GSH levels, attenuated neuronal death, and improved motor performance [137]. In addition, extracts from nature, such as prokineticin-2 from black mamba venom and frog skin, can promote ACSL4 ubiquitination and degradation to alleviate ferroptosis and protect mitochondria and neurons from damage [149]. A compound derived from Polygonum, Polydatin, enhanced the activity of GPX4 more than Fer-1, can reverse TBI-induced iron deposition, lipid peroxidation, and the expression of genes associated with ferroptosis response [150]. The new strategies and related drugs for CNS treatment can reduce ferroptosis and induce cascade reactions, thus finally reaching neuron reconnection and functional reestablishment.

#### Alzheimer’s disease

The incidence of neurodegenerative diseases, including Alzheimer’s disease (AD), Parkinson’s disease (PD), multiple sclerosis (MS), Huntington’s disease (HD), etc. grows with the increasing average life expectancy. However, the existing therapeutic interventions are still limited to curing these diseases, imposing an enormous burden on society [151]. Recently, mounting evidence has indicated the involvement of ferroptosis in neurodegenerative diseases, particularly in AD. The pathological changes in AD patients are typically characterized by senile plaques (SP) formed by the extracellular deposition of β-amyloid (Aβ) and neurofibrillary tangles (NFTs). Accumulation of SP and NFT can lead to neuronal atrophy and loss, resulting in neurological dysfunction [152]. Early studies have shown that iron accumulation is involved in the progression of AD, and that ferroptosis inhibitors have a protective effect in cellular or animal models of AD [153, 154].

Abnormalities in iron metabolism have serious implications for neuronal degenerative changes and brain dysfunction in AD. Iron accumulation and overload can promote Aβ and Tau deposition in the CNS and ferroptosis in microglia [155, 156]. Elevated ferritin, impaired GSH metabolism and iron imbalance have been observed in AD [157]. Ferroptosis-related genes STAT1, BCL2L11, and TFRC may be potential biomarkers for diagnosis and therapeutic monitoring of AD [158]. In addition, downregulation of FTH1 and SAT1 was found to be the main cause of astrocytes ferroptosis. Activation of ferroptosis in astrocytes may contribute to the pathophysiological process as well as emotional and cognitive disorders of AD by scRNA-seq analysis Dang et al. [159].

In terms of drug development for AD, some new drugs have shown strong antiferroptosis effects. Imidazolylacetophenone oxime ester was initially screened as a promising new drug for the effective treatment of AD with clear BBB penetration and no cytotoxicity, which effectively protects against ferroptosis-induced neuronal cell damage [160]. The ketogenic diet (KD) can downregulate TfR1, upregulates FTH1, and elevates the xCT/GPX4 axis to restrict iron dyshomeostasis and alleviate oxidative stress. Cognitive deficits, amyloid deposition and Tau hyperphosphorylation were effectively prevented in C57/BL6 mice in the KD group [161]. Since there are still limited studies, research on the alterations caused by the effects of ferroptosis in different types of cells during neurodegenerative processes will help to better develop target drugs.

## Ferroptosis in organ fibrosis diseases

Following acute and chronic injury, the uncontrolled regulation of inflammation in tissues causes excessive ECM deposition and fibrosis, which irreversibly destroys the structure and function of tissues and organs, resulting in organ failure and high mortality [5]. Fibrotic diseases can occur in multiple organs, including the heart, liver, kidneys, and lungs. The incidence and mortality of fibrotic disease are increasing globally; however, antifibrotic drugs applied to these diseases have shown limited effects [162]. Therefore, identifying the common mechanisms underlying the pathogenesis of fibrosis in different organs is critical.

**Fig. 4:**
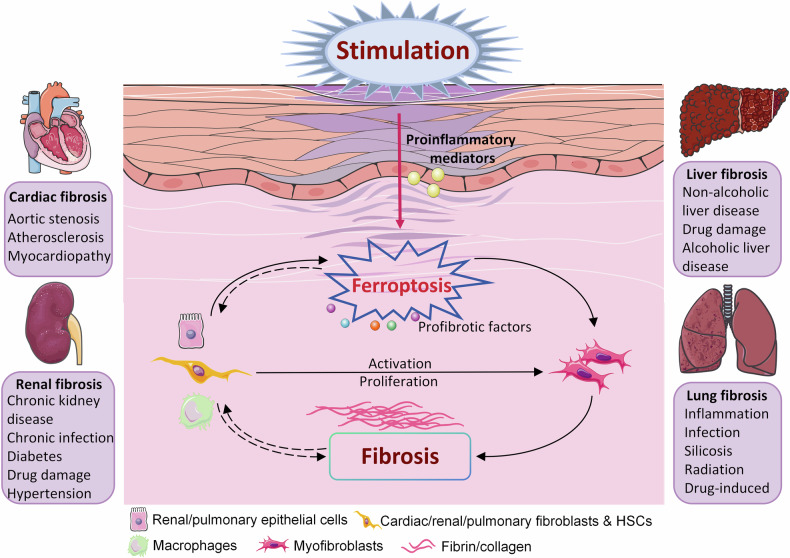
Schematic diagram of mechanisms and cellular events involved in organ fibrosis. The proinflammatory mediators are released after various stimulation, inducing ferroptosis in targeted cells. Multiple types of cells are recruited to release biological mediators, including chemokines and cytokines. Subsequently, fibroblasts and quiescent hepatic stellate cells are activated and transformed into myofibroblasts, which leads to tissue fibrosis.

Fibrosis is characterized by parenchymal cell injury, persistent inflammation, excessive ECM deposition, and myofibroblast activation. Parenchymal cell death is generally the essential step in fibrosis [163]. Inflammation, oxidative stress, and iron overload are intricately linked to ferroptosis in fibrotic diseases, suggesting that ferroptosis is an essential mechanism for regulating fibrosis. Ferroptosis in the parenchymal tissue cells of tissues exacerbates fibrosis progression, whereas regulating ferroptosis in myofibroblasts ameliorates fibrosis. Figure 4 demonstrates the potential link between ferroptosis and fibrotic progression in the pathogenesis of fibrotic diseases in different organs. We summarize the associations of ferroptosis with various fibrotic diseases to provide insights into the targeting of specific cells, metabolic pathways, key molecules, and potential therapeutic agents in fibrosis.

### Myocardial fibrosis

#### Chronic cardiovascular disease

Myocardial fibrosis is a pathological characteristic of several chronic cardiovascular diseases (e.g., myocardial infarction, heart failure, and cardiomyopathy) and is caused primarily by the differentiation of activated cardiac fibroblasts into myofibroblasts and collagen type I and III secretions [164]. In response to injury, cardiac fibroblasts proliferate and differentiate into myofibroblasts via a process mediated by TGF-β, endothelin-1, and angiotensin-1 [165]. Structural remodeling and functional impairment associated with myocardial fibrosis exacerbate arrhythmias and sudden cardiac death.

Current therapeutic approaches remain limited for myocardial fibrosis. Ferritin deposition occurs in numerous cardiovascular diseases, suggesting a close correlation between ferroptosis and cardiac fibrosis. Moreover, mitochondria-dependent ferroptosis occurs in adriamycin-induced myocardial fibrosis, and the ferroptosis inhibitor Fer-1 effectively reduces fibrosis severity [166, 167]. In the early stages of myocardial infarction, the Nrf2/Hmox1 pathway leads to iron overload and ferroptosis [168]. Mixed lineage kinase 3 (MLK3) signaling induces ferroptosis-related myocardial fibrosis in mice with advanced chronic heart failure by regulating the NF-κB/NLRP3 and JNK/p53 signaling pathways to aggravate oxidative stress [169]. Research suggests that autophagy-dependent ferroptosis promotes the progression of heart failure and cardiac remodeling via the TLR4-NOX4 pathway [170].

Interventions targeting ferroptosis may be promising strategies for treating myocardial fibrosis. Myocardial fibrosis induced by exposure to ionizing radiation can be inhibited by etomidate via the Nrf2 pathway, which inhibits cardiomyocyte ferroptosis [171]. By modulating the NLRP3 and MyD88 signaling pathways, empagliflozin can effectively control fibrosis by inhibiting ferroptosis and ameliorating inflammation in cardiac tissues [172]. In stress overload-induced myocardial fibrosis, elabela and Fer-1 alleviate late concomitant myocardial fibrosis via the IL-6/STAT3/GPX4 signaling pathway [173]. Similarly, xCT reduces angiotensin-induced cardiac fibrosis and pathological myocardial remodeling. Numerous natural herbal plant extracts, including fisetin, Rhodiola rosea extracts, silymarin and salicin, and Astragalus saponin IV [174–176], can protect the myocardium by targeting ferroptosis and are potentially effective and safe drugs.

#### Diabetic cardiomyopathy

Unlike unusual heart diseases, diabetic cardiomyopathy (DCM) is a nonvascular cardiac structural and functional disorder induced by an HG environment in a metabolically disordered state and is often accompanied by abnormal myocardial fibrosis and cardiac remodeling [177]. The incidence of myocardial ischemia in patients with diabetes is more than twice that in the normal population, and the risk of heart failure due to DCM is as high as 74% [178]. Cardiomyocytes from patients with diabetes are highly susceptible to lipid peroxide accumulation and redox dysregulation, which are highly correlated with the intrinsic mechanism of ferroptosis.

Myocardial injury in diabetic mice is associated with mitochondria-dependent ferroptosis, resulting in ROS accumulation and the downregulation of antioxidant enzymes [179]. Ghosh et al. [180] reported that GSH levels are reduced and the ROS levels are elevated in diabetic rat cardiomyocytes. The level of type III collagen, a marker of myocardial fibrosis, is significantly increased in an iron-overloaded rat model of type 1 diabetes mellitus [181]. During the pathogenesis of DCM, a high glycemic state results in increased intracellular lipid accumulation and lipotoxicity, which are highly susceptible to induced ferroptosis [182]. Owing to the pathophysiological process of DCM, increasing the anti-inflammatory and antioxidant capacities of cells is essential for effective treatment. NRF2 exerts antiferroptotic and antifibrotic functions to protect damaged pancreatic islet b-cells in DCM [183], and its activator, rutin, effectively controls central myofibrosis progression in diabetic mice [184]. Moreover, the NRF2/KEAP1/ARE and NRF2/FPN1 signaling pathways are key mechanisms of myocardial injury in DCM [185]. Lipid peroxidation and ferroptosis inhibitors can prevent aberrant myocardial remodeling in DCM by affecting the Fenton response and endoplasmic reticulum stress [186]. However, DCM still requires the development of more precise targeted therapies, and its connection to ferroptosis should be further explored.

### Liver fibrosis

Chronic injury occurs in alcoholic liver disease, nonalcoholic steatohepatitis, and chronic hepatitis B and C virus infections, ultimately leading to hepatic fibrosis [187]. The activation of hepatic stellate cells (HSCs), including transdifferentiation into myofibroblasts and excessive ECM deposition, is a critical stage of hepatic fibrosis [188]. As a clinically recommended treatment, liver transplantation has limited applications. Therefore, elucidating the pathogenic mechanism by targeting HSC activation inhibition or inducing activated HSC death could support liver fibrosis treatment and prevention.

Recent studies have focused on the bidirectional regulatory roles of ferroptosis in the progression and attenuation of liver fibrosis. Livers in mice fed a high-iron diet developed fibrosis, which was alleviated by the ferroptosis inhibitor ferrostatin-1 [189, 190]. Conversely, targeting ferroptosis in activated HCSs inhibits hepatic fibrosis. In a CCL4-induced mouse model of hepatic fibrosis, HSCs in the GSH depletion state, with low SLC7A11/xCT expression and E3 ubiquitin ligase TMCP26 overexpression, triggered ferroptosis, while fibrosis was effectively alleviated [191, 192]. These findings imply a critical relationship between hepatic fibrosis and iron concentration, and that excessive or insufficient iron concentration may result in hepatic fibrosis. However, precisely targeting activated HCSs to induce ferroptosis has an effect on liver fibrosis treatment.

Targeting specific hepatocytes with effective antifibrotic strategies and drugs is the key to treating liver fibrosis. In addition to the BRD7-P53-SLC25 A28 axis, ELAV-like protein 1 (ELAVL1) and zinc finger protein 36 (ZFP36) are promising targets for hepatic fibrosis prophylaxis [193]. Additionally, m6A modification (YTHDF1)-induced autophagy (BECN1)-associated ferroptosis may be a promising target for treating liver fibrosis [194]. Sorafenib can trigger ferroptosis in HSC via HIF-1α/SLC7111 signaling [195], and artemether [196], artesunate [197], chrysophanol [198], magnesium isoglycyrrhizinate [199], and berberine [200] are potential anti-hepatic fibrosis agents. Thus, inducing ferroptosis in HSCs may be a viable strategy for treating and preventing hepatic fibrosis; however, more information is needed to selectively induce ferroptosis in HSCs with minimal impact on healthy hepatocytes.

### Renal fibrosis

Renal fibrosis is defined as damage to the intrinsic cells within the renal parenchyma and transdifferentiation resulting in excessive deposition of the fibrotic matrix. It is a common endpoint in most chronic and progressive kidney diseases [201]. Renal fibrosis includes tubulointerstitial fibrosis and glomerulosclerosis, which lead to structural destruction and loss of kidney function. Terminal renal fibrosis leads to renal failure and requires dialysis or renal transplantation.

Ferroptosis is involved in renal fibrosis progression following tubular injury [202]. In the renal cortex of patients with chronic kidney disease (CKD), GSH expression is reduced, whereas oxidative stress and ROS levels are elevated [203]. Ferroptosis has been observed in animal models of various CKDs [202], unilateral ureteral obstruction [204], and ischemia/reperfusion injury [205]). Ferroptosis in renal tubular epithelial cells promotes the secretion of pro-fibrotic mediators such as TGF-β, CTGF, and PDGF, which regulate the EMT pathway to promote renal fibrosis [204]. Research has shown that, in contrast to ferroptosis inducers that exacerbate renal injury and fibrosis progression, ferroptosis inhibitors, such as liproxstatin-1 [204], nobiletin [206], tectorigenin [207], tocilizumab [208], rhein [209], and formononetin [210], can preserve renal injury by controlling lipid peroxidation and GSH depletion. Renal tubular epithelial cell ferroptosis and fibrosis in folate-induced AKI can also be effectively treated with roxadustat (FG-4592) [211]. Herbal components, such as quercetin [212], umbelliferone [213], ginkgolide B [214], and platycodin D [215], effectively ameliorate oxidative stress injury associated with ferroptosis in diabetic kidney disease. Thus, inhibiting ferroptosis is beneficial for tissue remodeling and protection against fibrosis following renal injury. However, future studies are needed to elucidate the role of ferroptosis in exerting therapeutic effects.

### Pulmonary fibrosis

Pulmonary fibrosis (PF) is commonly a consequence of infection, toxicity, and radiation. Idiopathic pulmonary fibrosis (IPF) is a complication of autoimmune diseases such as scleroderma [216], and is pathologically characterized by alveolar structure destruction, lung fibroblast proliferation, and ECM deposition [217]; however, its pathogenesis remains unclear. Currently, the intratracheal administration of bleomycin is the most common used experimental animal model of PF. Iron overload and oxidative stress imbalance occur in the lungs of patients with idiopathic IPF and in a mouse model of bleomycin-induced PF [218, 219]. Ferroptosis-related genes in the bronchoalveolar lavage fluid of patients with IPF were analyzed via bioinformatic characterization, which suggested the potential of ferroptosis in PF therapy [220]. Moreover, activating ferroptosis-related phenomena are activated in patients with radiation-induced lung fibrosis [221, 222], paraquat-induced PF [223], and in SARS-CoV-2 [224] infection.

The iron chelator DFO, combined with liproxstatin-1, suppresses ferroptosis to prevent PF induced by bleomycin and lipopolysaccharides [225]. Similarly, Fer-1 effectively inhibits lipid peroxidation and increases GPX4 expression for therapeutic benefit [226]. Conversely, erastin- and TGF-β1-treated lung fibroblasts presented increased activation and differentiation and accelerated the fibrosis process [227]. The methylation regulator Uhrf1 was upregulated in a PF mouse model and promoted pulmonary ferroptosis via epigenetic repression of the GPX4 and FSP1 genes [228]. Natural extracts that target ferroptosis may be promising therapeutic agents for treating PF. Taxifolin [229], *Tripterygium wilfordii* Hook.f. [223], and virofree [224], can attenuate silica-induced PF, paraquat-induced acute lung injury, and SARS-CoV-2 infection, respectively, in patients with fibrosis in vivo and in vitro by modulating iron metabolism and inhibiting ferroptosis. The preclinical mechanism of action of natural compounds that target ferroptosis for treating PF must be fully elucidated. More extensive preclinical studies and clinical trials are required to determine the optimal doses, efficacies, and safety of these compounds.

## Emerging techniques for designing ferroptosis-targeting therapies

The efficacy of multiple drugs targeting ferroptosis has been extensively studied in various fibrotic diseases on the basis of cellular and animal models (summarized in Table 2). Based on the established ferroptosis targets, significant efforts have been made in drug design. Existing clues suggest that inducers or activators of ferroptosis may improve fibrosis in different target cells in various disease environments, but the specific functions and mechanisms of drugs are still worthy of further exploration. High-throughput functional screening, automation, and artificial intelligence-based approaches can accelerate the development of targeted drugs. By improving the structure-function relationship, optimized Fer-1 molecules with better absorption, distribution, metabolism, and excretion profiles could enter preclinical and clinical trials [230]. Oncology is a leading and progressive approach for targeting ferroptosis and treating disease. Nanotechnology offers promising feasibility for inducing ferroptosis for the treatment of tumors, and nanomedicines based on nanomaterial delivery can generate ROS via the Fenton reaction [231]. The galactose-modified Fe304 nanodelivery system could be used as a novel chemodynamic therapy to eliminate senescent cells and promote wound healing [232]. The novel nanoscale secretory autophagosomes can restore dermal fibroblast function by inhibiting ferroptosis to promote diabetic wound healing [233].

**Table 2:** Drugs targeting ferroptosis for alleviating fibrotic diseases.

Organ	Drug	Cells and model	Effect on ferroptosis	Mechanism	Reference
Heart	Astragaloside IV	Murine model of AlC	Inhibit	Reduces oxidative stress; enhances Nrf2 signaling	[176]
	Dexmedetomidine	Rat model of MIRI	Inhibit	Activates SLC7A11/GPX4 axis	[242]
	Etomidate	Rat model of MIR	Inhibit	Activates Nrf2	[171]
	Empagliflozin	Cardiomyocyte & Murine model of DIC	Inhibit	Inhibits SGLT2 through NLRP3/MyD88-related pathways	[172]
	Fisetin	Cardiomyocyte & Rat model of DIC	Inhibit	Activates SIRT1/Nrf2 signaling pathway	[174]
	Histochrome	Cardiomyocyte & Rat model of MIRI	Inhibit	Activates Nrf2; increases GPX4 and free GSH	[243]
	Salidroside	Cardiomyocyte & Murine model of DIC	Inhibit	Prevents lipid peroxidation	[175]
Liver	Artesunate	HSC & Murine model of CCl_4_-LF	Induce	Triggers ferritinophagy	[197]
	Artemether	HSC & Murine model of CCl_4_-LF	Induce	Promotes p53 expression, promotes iron accumulation	[196]
	Berberine	HSC & Murine model of TAA-LF and CCl_4_-LF	Induce	Increases ROS level	[200]
	Erastin	HSC & Murine model of BDL-LF	Induce	Activates ferritinophagy	[193]
	Sorafenib	HSC & Murine model of CCl_4_-LF	Induce	Activates HIF-1a/SLC7A1 pathway	[240]
	Chrysophanol	HSC	Induce	Promotes ROS accumulation and lipid peroxidation	[198]
	Dihydroartemisinin	HSC & Rat model of CCl_4_-LF	Induce	Activates NCOA4 expression	[244]
	FGF21	Hepatocytes & murine model of iron overload-induced LF	Inhibit	HOI inhibition activates Nrf2 and inhibits HO-1	[245]
Kidney	Ferrostatin-1	Murine model of diabetic renal tubular injury	Inhibit	prevents ROS formation derived by NADPH oxidases	[32]
	Deferoxamine	TEC & rat model of 5/6 nephrectomy induced CKD; murine model of UUO-RF or IRI-RF	Inhibit	Reduces Fe deposition and prevents lipid peroxidation	[202]
	Liproxstatin-1	TEC & Murine model of UUO-RF	Inhibit	inhibits the secretion of the pro-fibrotic factors	[204]
	Tocilizumab mimotope	Murine model of UUO-RF	Inhibit	Downregulates the pro-fibrotic proteins; activates macrophage F4/80+ cells	[208]
	Nobiletin	Murine model of UUO-RF	Inhibit	Reduces oxidative stress and inflammation	[206]
	Tectorigenin	TEC & Murine model of UUO-RF	Inhibit	Inhibits Smad3 phosphorylation	[207]
	Roxadustat	Murine model of folic acid-induced kidney injury	Inhibit	Activates Akt/GSK-3β/Nrf2 pathway	[211]
Lung	Ferrostatin-1	Macrophage & Murine model of SiO_2_-induced PF	Inhibit	Inhibits lipid peroxidation	[246]
	Deferoxamine	Alveolar type ll cells; bronchial epithelial cells & Murine model of BLM-PF	Inhibit	Prevents iron deposition and mitochondrial dysfunction	[225]
	Liproxstatin-1	Murine model of radiation-induced PF	Inhibit	Suppresses TGF-β1 expression by the activating Nrf2	[221]

Numerous noncoding RNAs have been reported to play a role in regulating ferroptosis. Because PRDX3 is a newly identified marker of ferroptosis in chronic liver disease, its future application in disease treatment is promising [234]. Moreover, studies have been conducted to provide a theoretical basis for targeting the FSP1-dependent phase separation as an effective anticancer therapy. However, whether ferroptosis in specific cell types, such as hematopoietic stem cells, can be targeted to treat fibrosis more effectively remains a challenge.

## Summary and future perspectives

Significant progress has been made in our understanding of the pathological role of ferroptosis in repair and regeneration processes. Ferroptosis primarily acts as a stimulator and exacerbator of the post-injury inflammatory response, promoting tissue ROS release and lipid peroxide accumulation to further influence skin trauma repair, group dysmorphic remodeling between bones and joints, and inflammatory fibrosis in multiple organ types. GPX4, ACSL4, FSP1, and various transcription factors play key regulatory roles in ferroptosis. If aberrant effector cells involved in tissue repair and remodeling are targeted and induced undergo ferroptosis, thereby achieving control of the downstream inflammatory response, a poor prognosis can be effectively prevented.

For the challenges in studying ferroptosis in the development of regeneration and fibrosis, at present, there are still some perspectives worth pondering. Firstly, it remains unclear whether ferroptosis is the dominant PCD type or how different types of PCD promote or inhibit each other during injury and repair. In the future, the interrelationship and dominance of ferroptosis with other PCDs in the pathological process need to be further determined to elucidate the mechanisms of multicellular regulation of death forms. Secondly, based on the four stages of damage repair: hemostasis, inflammation, proliferation, and remodeling, finding biomarkers in different phages needs further investigation, especially cells or differentially expressed genes that play key roles in the transition stage from late inflammation to proliferation and remodeling. Designing specific knockouts for these differences and combination with novel biomaterials to develop targeted antifibrotic drugs is also a good approach.

Lastly, the results of interventions targeting different ferroptosis pathways vary, and gaps remain in our understanding of the processes involved. Therefore, identifying noninvasive biomarkers that regulate ferroptosis in animal models and human diseases while clarifying the optimal interventions, timing of interventions, and patient populations is important. Moreover, interventions that act on two or three ferroptosis pathways may increase the likelihood of therapeutic success; however, safety issues must be further explored and discussed.

## Data Availability

All data included in this study are available upon request by contact with the corresponding author.
